# Pervasive Phylogenomic Incongruence Underlies Evolutionary Relationships in Eyebrights (*Euphrasia*, Orobanchaceae)

**DOI:** 10.3389/fpls.2022.869583

**Published:** 2022-05-27

**Authors:** Phen Garrett, Hannes Becher, Galina Gussarova, Claude W. dePamphilis, Rob W. Ness, Shyam Gopalakrishnan, Alex D. Twyford

**Affiliations:** ^1^GLOBE Institute, University of Copenhagen, Copenhagen, Denmark; ^2^Institute of Evolutionary Biology, School of Biological Sciences, University of Edinburgh, Edinburgh, United Kingdom; ^3^Natural History Museum, University of Oslo, Oslo, Norway; ^4^Botany Department, Faculty of Biology and Soil Science, St Petersburg State University, St Petersburg, Russia; ^5^Tromsø University Museum, University of Tromsø, Tromsø, Norway; ^6^Department of Biology and Huck Institutes of the Life Sciences, The Pennsylvania State University, University Park, PA, United States; ^7^Department of Biology, University of Toronto Mississauga, Mississauga, ON, Canada; ^8^Royal Botanic Garden Edinburgh, Edinburgh, United Kingdom

**Keywords:** phylogeny, discordance, *Euphrasia*, taxonomic complexity, plastid, whole-genome sequencing

## Abstract

Disentangling the phylogenetic relationships of taxonomically complex plant groups is often mired by challenges associated with recent speciation, hybridization, complex mating systems, and polyploidy. Here, we perform a phylogenomic analysis of eyebrights (*Euphrasia*), a group renowned for taxonomic complexity, with the aim of documenting the extent of phylogenetic discordance at both deep and at shallow phylogenetic scales. We generate whole-genome sequencing data and integrate this with prior genomic data to perform a comprehensive analysis of nuclear genomic, nuclear ribosomal (nrDNA), and complete plastid genomes from 57 individuals representing 36 *Euphrasia* species. The species tree analysis of 3,454 conserved nuclear scaffolds (46 Mb) reveals that at shallow phylogenetic scales postglacial colonization of North Western Europe occurred in multiple waves from discrete source populations, with most species not being monophyletic, and instead combining genomic variants from across clades. At a deeper phylogenetic scale, the *Euphrasia* phylogeny is structured by geography and ploidy, and partially by taxonomy. Comparative analyses show Southern Hemisphere tetraploids include a distinct subgenome indicative of independent polyploidy events from Northern Hemisphere taxa. In contrast to the nuclear genome analyses, the plastid genome phylogeny reveals limited geographic structure, while the nrDNA phylogeny is informative of some geographic and taxonomic affinities but more thorough phylogenetic inference is impeded by the retention of ancestral polymorphisms in the polyploids. Overall our results reveal extensive phylogenetic discordance at both deeper and shallower nodes, with broad-scale geographic structure of genomic variation but a lack of definitive taxonomic signal. This suggests that *Euphrasia* species either have polytopic origins or are maintained by narrow genomic regions in the face of extensive homogenizing gene flow. Moreover, these results suggest genome skimming will not be an effective extended barcode to identify species in groups such as *Euphrasia*, or many other postglacial species groups.

## Introduction

Gene tree discordance is a pervasive feature of plant phylogenies, with numerous studies revealing diverse and conflicting topologies among loci within a genome (Stull et al., 2020; Rose et al., 2021; Wagner et al., 2021). While discordance is frequently seen as a barrier to species tree reconstruction and an impediment to taxonomic and systematic research, characterizing discordance can provide major insights into evolutionary processes. For example, discordance at deep phylogenetic scales can be indicative of hybridization that has promoted major species radiations (Marques et al., 2019), while discordance at shallow phylogenetic scales can reveal contemporary population processes, such as the balance between drift, gene flow, and selection in the maintenance of genetic variation (Lee-Yaw et al., 2019). Phylogenetic discordance is likely to be most prevalent in certain plant groups, particularly those characterized by hybridization, polyploidy, and/or recent (often postglacial) speciation (Squirrell et al., 2002; Wagner et al., 2021). This includes taxonomically complex plant groups (*sensu* Ennos et al., 2005), such as sedges (*Carex*), willows (*Salix*), and *Epipactis* orchids, where discrete species are often hard to define.

Phylogenomic studies of taxonomically complex groups are fraught with difficulties, with these poorly studied groups often lacking genomic resources, such as reference genomes, and with bioinformatic issues associated with the analysis of polyploids (Brandrud et al., 2020). Despite these challenges, the emergence of low-cost genomic sequencing, coupled with bioinformatic tools for the analysis of large and complex phylogenomic data sets, makes these issues ever more tractable. For example, genomic data can be generated from expertly determined herbarium samples even if the DNA shows evidence of degradation (Bakker et al., 2016), providing a phylogenetic context and taxonomic framework for interpreting relationships in taxonomically complex groups. Moreover, even low-coverage genomic data, such as genome skimming (Straub et al., 2012), can be useful for recovering multiple independent subcellular genomes, such as the plastid, mitochondrial, and nuclear ribosomal DNA (nrDNA). Studying haploid organellar genomes, as well as nrDNA where repeats are generally expected to be homogenized within an individual *via* concerted evolution (Xu et al., 2017), circumvents many issues associated with polyploid phylogenetics and provides an opportunity to compare phylogenetic signal between genomes with conflicting modes of inheritance (Rieseberg and Soltis, 1991). If nuclear genomic data can also be recovered this may facilitate more detailed characterization of a groups’ evolutionary history, such as investigating the evolutionary history of the two or more composite “subgenomes” in allopolyploids (Chen et al., 2020). Despite this promise, few studies to date have investigated phylogenetic discordance in taxonomically complex plant groups (though see Brandrud et al., 2020).

The genus *Euphrasia*, commonly known as eyebrights, are a diverse group comprising 273 annual and perennial species, with a bipolar distribution. Across the genus, there are various ploidy levels, from diploids to dodecaploids, with multiple independent polyploidy events from a base chromosome number of 11 (Gussarova et al., 2008). All species are hemiparasites that attach to, and feed from, a broad range of plant hosts (Yeo, 1964; Brown et al., 2021). The genus is perhaps most renowned for its taxonomic complexity, particularly in Europe (French et al., 2008). The postglacial radiation in northern Europe includes numerous closely related taxa that are extremely challenging to separate based on morphology (Yeo, 1978) or with DNA barcoding (Wang et al., 2018). The small stature of these plants (frequently <10 cm tall) and their phenotypic plasticity (Karlsson, 1984; Zopfi, 1997; Brown et al., 2020), coupled with many traits demonstrating population-level rather than species-level differences due to limited gene flow as a consequence of their selfing or partly selfing mating system (French et al., 2005), all further confound species identification. Furthermore, species show extensive interfertility and a large array of natural hybrids have been recorded in the wild (Stace et al., 2015). The most extensive taxonomic issues have been noted from tetraploid species, though issues are present at all ploidy levels.

Previous studies of the genus have successfully confirmed the monophyly of *Euphrasia* and resolved some broad-scale relationships, though have also faced significant challenges. In terms of broad-scale studies, the largest global phylogeny to date used three plastid regions and the internal transcribed spacer (ITS) of nrDNA generated for 51 species (Gussarova et al., 2008). This study recovered phylogenetic relationships relating to broad-geographic regions and by ploidy. However, for both data sets, there were issues with unresolved species-level relationships, with these particularly pronounced for European taxa in the plastid phylogeny, which were largely unresolved. Moreover, the underlying evolutionary processes shaping the topology were hard to infer with few gene regions, and it may be that nrDNA homogenization or loss of ancestral plastid variants could cause these global phylogenies to deviate from the expected nuclear species tree. At a smaller geographic scale, population genomic sequencing of 18 samples of British *Euphrasia*, with a particular sampling focus of co-occurring species on the small Scottish island of Fair Isle, found that species share extremely similar plastid DNA sequences (>99.8% similarity based on whole plastid genomes), with phylogenetic relationships not closely tracking species boundaries and only weakly clustering by geography (Becher et al., 2020). Diploids and tetraploids were characterized by highly divergent nrDNA arrays (10.8% divergence in ITS sequences), though species-level relationships remained unclear. Here, neither plastid DNA nor nrDNA closely followed the pattern observed across the nuclear genome in these samples.

In this study, we use genomic data to investigate phylogenetic discordance in taxonomically complex *Euphrasia*. To do this we adopt a two-stage strategy. First, we study the enigmatic relationships of postglacial northern European *Euphrasia* species, particularly those present in Britain. We build on a number of previous genetic studies (French et al., 2008; Gussarova et al., 2008; Wang et al., 2018; Becher et al., 2020), generating new sequence data and re-analyzing previous sequences. This microevolutionary focus, aimed at using dense species sampling and the use of multiple individuals per species, allows us to investigate: (1) phylogenetic relationships and the evidence for recurrent colonization of the British Isles from continental Europe, (2) the nature of species differences and whether hybridizing British *Euphrasia* species are monophyletic. Secondly, we study a sparser sample of diverse species from across the *Euphrasia* phylogeny, with the aim of investigating broader scale macroevolutionary processes. In particular, we look test: (3) whether there is evidence of phylogenetic discordance deep in the *Euphrasia* phylogeny, (4) whether discordance may be a consequence of more complex genome evolutionary dynamics in newly sequenced polyploids. Our approach involves diverse herbarium material used for genomic sequencing, and comparative genomics, to document phylogenetic discordance between independent subcellular genomes and genomic regions (the nuclear genome, nrDNA arrays, plastid genomes).

## Materials and Methods

### Plant Material, DNA Extraction, and Genomic Sequencing

Our phylogenomic analyses included a total of 58 samples, 56 samples from 36 *Euphrasia* species, and two outgroup species, *Bartsia alpina* L. and *Neobartsia chilensis* Uribe-Convers & Tank. This material included a combination of newly sequenced samples and previously generated data, with full sample information provided in Supplementary Table S1. To investigate global evolutionary relationships, broad-scale phylogenetic conflict, and diversity in modes of ploidy across the genus, we selected a shallow sample of taxa that maximized representation of geographic regions (including Northern and Southern Hemisphere taxa), and to capture taxonomic diversity and anticipated evolutionary divergence times. To investigate phylogenetic relationships and species cohesion in postglacial northern European *Euphrasia*, particularly in Britain, we used available sequences from a range of different studies to maximize species coverage, and where possible to include multiple individuals per species.

For the broad-scale analysis, we sequenced herbarium material from 17 *Euphrasia* species. Herbarium samples were obtained from the University of Copenhagen (C), the Royal Botanic Garden Edinburgh (E), and Oslo University Herbarium (O). The herbarium samples spanned 1861–2019 and included a broad range of collection localities covering 15 countries including Canada, New Zealand, and Sweden (Supplementary Table S1). DNA was extracted from samples using the Qiagen DNEasy Plant Extraction kit. Extractions were quantified using the Qubit 2.0 Fluorometer (Applied Biosystems).

Library building was performed using Copenhagen University’s EvoGenomics’ in-house BEST protocol (Carøe et al., 2018), which is a PCR-based, short-insert library preparation method designed to maximize historical DNA potential by accounting for low extraction yields. Purification steps were used both at the extraction stage and the library building stage and included both SPRI magnetic beads (Beckman Coulter) and membrane filter MinElute PCR Purification spin columns (Qiagen). Subsequently, Illumina dual indexes (8 bp) were used to facilitate multiplexing of samples. Sequencing was outsourced to NovoGene EU, using the NovaSeq 6,000 with 150 bp paired-end (PE) sequencing.

For the analysis of phylogenetic relationships and monophyly in postglacial northern European *Euphrasia*, sequencing data for 41 individuals were sourced from three previous studies. First, we integrated data for 18 *Euphrasia* samples previously used in a population genomic study of British *Euphrasia*, with a focus on tetraploid species on Fair Isle, Scotland (Becher et al., 2020). This study generated a reference genome of the tetraploid species *E. arctica* (described below) and high-coverage short-read data for 17 additional *Euphrasia* samples. All 18 samples had nuclear SNPs called relative to the reference genome, and plastid genomes and nrDNA arrays assembled *de novo*. Second, low-coverage short-read sequencing data of 12 samples: 10 other British *Euphrasia*, one Austrian *Euphrasia*, and an outgroup *Bartsia alpina*, were available from a study characterizing the landscape of genomic repeats (Becher et al., 2021), with the raw data reanalyzed here and used for *de novo* assembly of plastid genomes and nrDNA, and mapping to the reference genome. Finally, we included short-read data for 11 previously unpublished samples (Twyford, Unpublished Data) where data was available on the Sequence Read Archive (SRA; SRR17976421 - SRR17976431). These represent 10 diverse *Euphrasia* taxa and an outgroup *Neobartsia chilensis*. These low-coverage genome skims were generated from NEB Ultra PCR-based libraries sequenced with 125 bp PE sequencing on the Illumina HiSeq 2500 or 150 bp PE sequencing on the Illumina NovaSeq 6000 at Edinburgh Genomics.

Our final data set included 31 samples collected in Britain, including multiple samples for: *E. anglica* Pugsley (2 samples), *E. arctica* Lange ex Rostr. (7), *E. confusa* Pugsley (2), *E. foulaensis* Towns. ex Wettst. (5), *E. micrantha* Rchb. (7) and *E. vigursii* Davey (2). These also included two putative hybrids (*E. confusa* x *E. foulaensis*, *E. arctica* x *foulaensis*) and two species of putative hybrid origin (*E. rivularis*, *E. vigursii*, Yeo, 1956). We also include a sample of ‘*Euphrasia fharaidensis*’, a UK endemic awaiting formal description (French et al., 2008). Herbarium material from all newly sequenced samples are lodged at E.

### Sequence Analysis

#### Plastid Genome Assembly and Curation

Plastid genomes were assembled for each sample *de novo*, using Novoplasty (Dierckxsens et al., 2017) or GetOrganelle (Jin et al., 2020). Most assemblies were circular, single-contig genomes, however where this was not the case assemblies were subject to additional curation. Specifically, any sample with a large deletion relative to other samples (more than 500 bp) had raw reads mapped back to the *E. arctica* reference plastid genome (Becher et al., 2020) using Geneious v11.1, and with coverage of putative deletions inspected by eye. Several regions for nine samples were then manually added to the plastid genome assemblies. Assembled plastid genomes were manually curated and edited to give a standard order of the large single copy (LSC), inverted repeat (IR), small single copy (SSC), and second copy of the IR, using Geneious. Newly assembled plastid genomes and previous plastids (Becher et al., 2020) were subsequently aligned using MAFFT (Katoh and Standley, 2013).

#### nrDNA Assembly and Curation

nrDNA arrays were assembled using Novoplasty with the expected assembly size set to 9,000–20,000 bp and using a 1,380 bp seed sequence of the nrDNA cluster, obtained from a run of the RepeatExplorer pipeline (Novák et al., 2013). Variable results were produced by the assembler, with some samples having fully assembled circularized arrays and others having multiple, overlapping contigs. Ambiguous sites were coded with standard nucleotide ambiguity codes, with these sites potentially representing divergent ribotypes maintained within individuals, or uncertainty in the underlying sequencing or assembly. To avoid assembly issues or problems aligning the highly variable external transcribed spacer (ETS), the assemblies were subsequently trimmed to the ~5.8Kb nrDNA coding region (comprising 18S, ITS1, 5.8S, ITS2, 26S, termed the nrDNA array herein). nrDNA arrays were subsequently aligned using MAFFT.

#### Nuclear Genome Resequencing

Paired sequence reads were mapped to the tetraploid *E. arctica* genome (Becher et al., 2020). This reference genome assembly was produced using high-coverage Illumina data in conjunction with low-coverage Pacific Bioscience data and spans 823 Mb of the ~1.15Gb genome. Characterization of the *E. arctica* genome has shown it to be an old allotetraploid with divergent subgenomes, one of which is closely related to extant British diploid taxa (Becher et al., 2020).

Reads were mapped using the PALEOMIX (Schubert et al., 2014) pipeline, apart from the 18 samples from the study of Becher et al. (2020) which were already aligned and available as a BAM file. The PALEOMIX pipeline is especially designed for the mapping and initial processing of degraded DNA, making it particularly suitable for the herbarium samples included in this study. Raw reads were initially trimmed for ambiguous and low-quality bases at the ends of reads (N, or base quality less than 2). Subsequently, adapter sequences were identified and excised from the 3′ ends of the short reads using AdapterRemoval (v2.2.2) (Schubert et al., 2014). As part of the adapter trimming, read pairs that overlapped by more than 10 bp were merged, and any reads shorter than 25 bp were discarded. The adapter trimmed reads were mapped to the reference genome using the bwa aln algorithm (v0.7.15; Li and Durbin, 2010), with seeds disabled to allow better matches for degraded DNA. Finally, reads aligning to the reference genome with mapping quality less than 30 were discarded from downstream analyses.

Previous analyses of genome resequencing data for 14 British tetraploid and 4 British diploid samples identified a set of 3,454 conserved scaffolds longer than 1 kb, that have coverage consistent with diploid-level mapping depth across all individuals (Becher et al., 2020). In total, these scaffolds represent 46 Mb of the genome. These were proposed to represent disomically inherited nuclear regions homologous across ploidy levels, and belonging to a shared subgenome. Here, we selected these scaffolds for downstream phylogenomic analysis between taxa of varying or unknown ploidy. These scaffolds can be directly compared across ploidy levels and used in conventional phylogenetic packages suitable for diploid taxa, though with the caveat that we may undersample duplicated regions. Sequences for the conserved scaffolds were extracted from the mapping data for each sample based on the scaffold coordinates in the reference genome. FASTA files of consensus sequences were produced with Angsd (Korneliussen et al., 2014) using a quality threshold set at bp-site 3X coverage. Data for each scaffold was filtered using custom Python scripts to remove any samples without representative consensus sequences. Any scaffold with less than three samples represented by consensus sequences were also removed. In addition to focused phylogenomic analyses of the conserved scaffolds, we also investigated analyses of the complete nuclear genome directly from the raw sequence reads (described below).

### Phylogenetic Analyses

Phylogenetic analyses were performed independently for plastid genomes, nrDNA arrays, conserved nuclear scaffolds and the complete set of sequence reads. For each data set, phylogenies were annotated with available ploidy and geographic information. Ploidy information came from Metherell and Rumsey (2018) and Becher et al. (2021) for British samples and Gussarova et al. (2008) for other taxa. Ploidy information was available for 35 individuals, with missing information for many newly sequenced non-British samples. Geographic areas were annotated on trees following the areas defined by Gussarova et al. (2008).

#### Plastid Genome

Phylogenetic analyses of the complete plastid genome sequences were performed using IQTree 2 (Minh et al., 2020) with the best evolutionary model inferred using model fitting and model assessment based on Bayesian Information Criterion. Maximum likelihood trees were obtained using the selected best evolutionary model, and branch support was inferred *via* 1,000 rapid bootstrap replicates. Trees were visualized with FigTree.

#### nrDNA

Partitioned phylogenetic analysis of the nrDNA arrays was performed in IQTree 2 to account for substitution rate variation (i.e., ITS has a higher substitution rate than other nrDNA regions). Maximum likelihood trees were obtained in IQTree 2, and branch support was inferred *via* 1,000 rapid bootstrap replicates.

#### Nuclear Genome Resequencing

Phylogenetic analyses were performed on each of the conserved nuclear scaffolds separately, with these then being used to build a putative species tree. IQTree 2 was first used to build Maximum Likelihood trees for each scaffold. Newick Utilities (Junier and Zdobnov, 2010) was used on each IQTree scaffold tree to collapse unsupported branches (bootstrap support, 10) before using Astral III (Zhang et al., 2018) to build a species tree from the suite of input scaffold trees, with each tree given equal weighting. DiscoVista (Sayyari et al., 2018) was used to compute and visualize the discordance between the species tree and each scaffold tree.

To investigate evolutionary relationships across the genome (not just in the subset of conserved nuclear scaffolds), and to further explore the utility of sequence data from our herbarium samples, we tested MASH (Ondov et al., 2016), an implementation of the MinHash approach to rapidly compute distances between strings. We analyzed the raw sequence reads for the 17 newly sequenced *Euphrasia* herbarium samples using default parameters, with the unrooted neighbor-joining tree visualized using FigTree.

#### Comparative Phylogenetics

Tanglegrams implemented in Dendroscope version 3.7.5 (Huson and Scornavacca, 2012) were used to detect discordance between the topologies of phylogenetic trees created using different genome partitions. Tanglegrams allow the comparison of rooted trees by rotating nodes to minimize perceived incongruence related to tree visualization. Our comparisons were between: (1) the nrDNA array and the plastid genome, (2) the plastid genome and the species tree from the conserved nuclear genome scaffolds, and (3) the nrDNA array and the species tree from the conserved nuclear genome scaffolds. The tanglegrams were generated with Dendroscope (v 3.7.5) and visualized in R (v 4.1.2) using packages dendextend (v1.15.2), phylogram (v2.1.0) and ape (v5.6-1).

### Genomic Analyses of Polyploidy

Previous genomic analyses of British *Euphrasia* inferred individual ploidy, and whether subgenomes are likely to be shared between individuals, based on sequencing coverage per genome scaffold relative to the *E. arctica* reference genome (Becher et al., 2020). In this previous analysis, while all samples had similar coverage across the conserved scaffold set (which are likely to be within a conserved subgenome present across diploid and tetraploid British *Euphrasia*), diploids had no (or very low) coverage in a large number of scaffolds restricted to the tetraploids. Here, we perform a similar mapping depth analysis across our global *Euphrasia* samples to understand whether genome structure is conserved across diverse species. The per-scaffold coverages were computed for each sample, retaining only scaffolds longer than 1 kb. In order to compare the samples within the study and to previously published data (Becher et al., 2020), the per-scaffold coverages were normalized by the average coverage across these scaffolds. Further, the analysis was restricted to ~10,000 scaffolds previously identified as conserved across the genus. Hierarchical clustering was performed on the resulting matrix of normalized per-scaffold coverages at these ~10,000 scaffolds. Coverages were then visualized per sample and per scaffold in a heatmap following Becher et al. (2020). To aid visualization samples were ordered based on relatedness inferred from hierarchical clustering of pairwise Manhattan distances between the samples’ mapping depth profiles (Becher et al., 2020).

## Results

We were able to recover nuclear genomic data from all 17 herbarium samples, with read counts averaging 276,825,100 per sample (range 241,730,838-312,518,834). These samples were combined with the 42 previously sequenced samples to investigate phylogenomic relationships of plastid genomes, nrDNA arrays, and the nuclear genome.

### Plastid Genome Diversity and Phylogenetic Relationships

We successfully assembled the plastid genome for 38 *Euphrasia* individuals and 2 outgroups and compared these to 18 previously assembled plastid genomes. Overall the assembly length was consistent across samples (range: 140,581–145,113 bp for *Euphrasia* species, up to 153,370 bp in *Bartsia alpina*), with a mean size of 144,792 bp across *Euphrasia* species. The average plastid genome GC content was 38.3%. Pairwise identity between *Euphrasia* samples was high at 99.1% and with 87.1% of sites identical across species, with these rising to 99.8 and 98.8%, respectively, across the 31 British samples. The final alignment of 56 *Euphrasia* and 2 outgroup plastid genomes was 157,898 bp in length.

**Figure 1:**
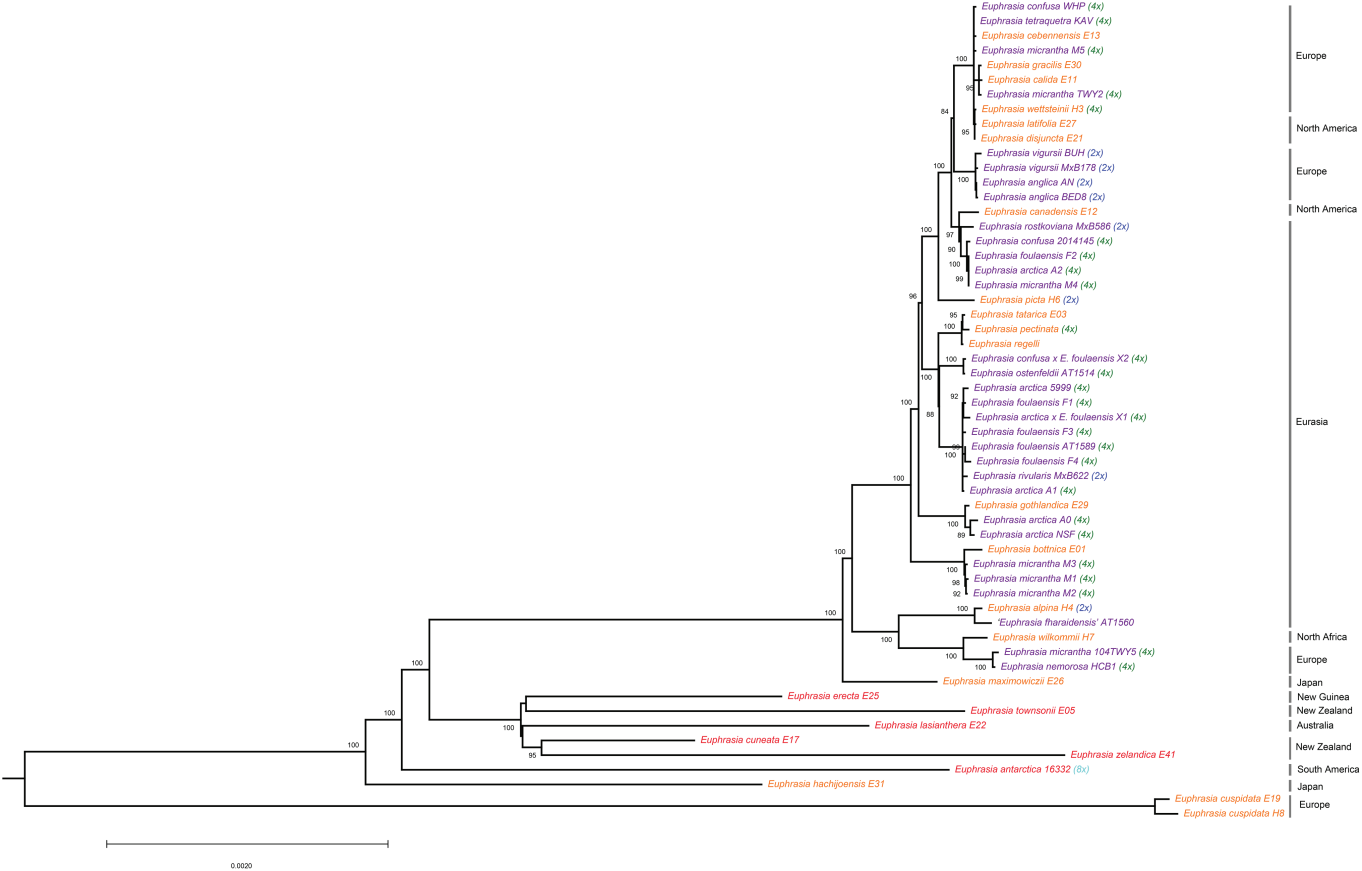
Phylogenetic relationships inferred from plastid genomes for global *Euphrasia* species. Phylogeny constructed based on an alignment of 157,898 sites, using a maximum likelihood analysis implemented in IQTree 2. Branches labeled with bootstrap support values over 85%. Phylogeny rooted on *Bartsia*, which is removed here for better visualization. Tip labels are colored by geography, with red being Southern Hemisphere, purple being UK and orange being European and Asian samples. Tips are also labeled by known ploidy, including diploids (2x, blue), tetraploids (4x, green) and octoploids (8x, light blue).

Phylogenetic analyses of plastid genomes revealed a deep split between clades broadly corresponding to Northern Hemisphere taxa, and Southern Hemisphere taxa plus samples from Japan (Figure 1). The only exception to this biogeographic split were two samples of the European species *E. cuspidata*, which were placed on a long branch separate from all other samples, consistent with it being a morphological distinct diploid taxon belonging to a separate taxonomic section. Within clades, there was significant phylogenetic complexity, with some patterns of relatedness representing geography, ploidy, or species identities, though many relationships are hard to explain. Within the poorly sampled clade of largely Southern Hemisphere species (represented by eight samples) species do not cluster by geography, with species sampled from the same country (such as New Zealand) separated on the tree.

Within the Northern Hemisphere clade, there is a lack of discernable overall phylogenetic structure and the tree is characterized by extremely short terminal branches. However, some individual or species-level patterns of plastid haplotype sharing and relatedness emerge. These include clusters corresponding to: three samples of *E. micrantha* from Fair Isle; related tetraploid *E. arctica* and *E. foulaensis* from Fair Isle; four diploid samples from *E. anglica* and *E. vigursii* from England; and a cluster of distinct Eurasian species *E. tatarica*, *E. pectinata*, and *E. regelii*. In contrast to these clusters, many more patterns of relatedness appear more complex, for example four other samples of *E. micrantha* (excluding the three samples that cluster) are largely spread throughout the wider Palearctic and Alps group. This pattern of individuals being scattered throughout the Northern Hemisphere clade is also seen with *E. arctica* and *E. foulaensis*.

Overall, the plastid phylogeny highlights the phylogenetic complexity present in the genus, with only weak clustering by geography, and with multiple samples within species not showing clear taxonomic coherence.

### nrDNA Phylogenetic Relationships

The nrDNA array was successfully assembled for all 38 samples except *E. gothlandica* and *E. micrantha* sample M4, which consistently failed. The new nrDNA assemblies were aligned with the 18 existing assemblies (Becher et al., 2020) to produce a conserved nrDNA alignment of 5,845 bp in length. Per sample conserved nrDNA array lengths varied between 5,571–5,828 bp across the alignment of 55 *Euphrasia* individuals and 2 outgroup samples. Pairwise sequence identity between *Euphrasia* individuals was 99.2, and 91.8% of sites were identical across the alignment. Total GC content was 54.3%. Most sites were unambiguously identified, with only 423 sites in the alignment (0.13% sites) coded as ambiguous. Across samples 21 individuals had no ambiguous sites (36.8%). These samples were either Southern Hemisphere taxa, all of which had no ambiguities (apart from octoploid *E. antarctica*), known Northern Hemisphere diploids (e.g., *E. alpina* and *E. vigursii*), or Northern Hemisphere species of unknown ploidy clustering with the diploids (*E. bottnica*, *E. canadensis*, see below). Exceptions to this finding are diploid *E. rostkoviana* and the putative diploid hybrid species *E. rivularis*, which had some ambiguous sites. All known tetraploid samples from the Northern Hemisphere had at least two ambiguous sites. Most ambiguous sites were not random in their position across the alignment and instead were common at sites segregating for two alleles, indicative of the retention of multiple nrDNA copies rather than assembly errors.

**Figure 2:**
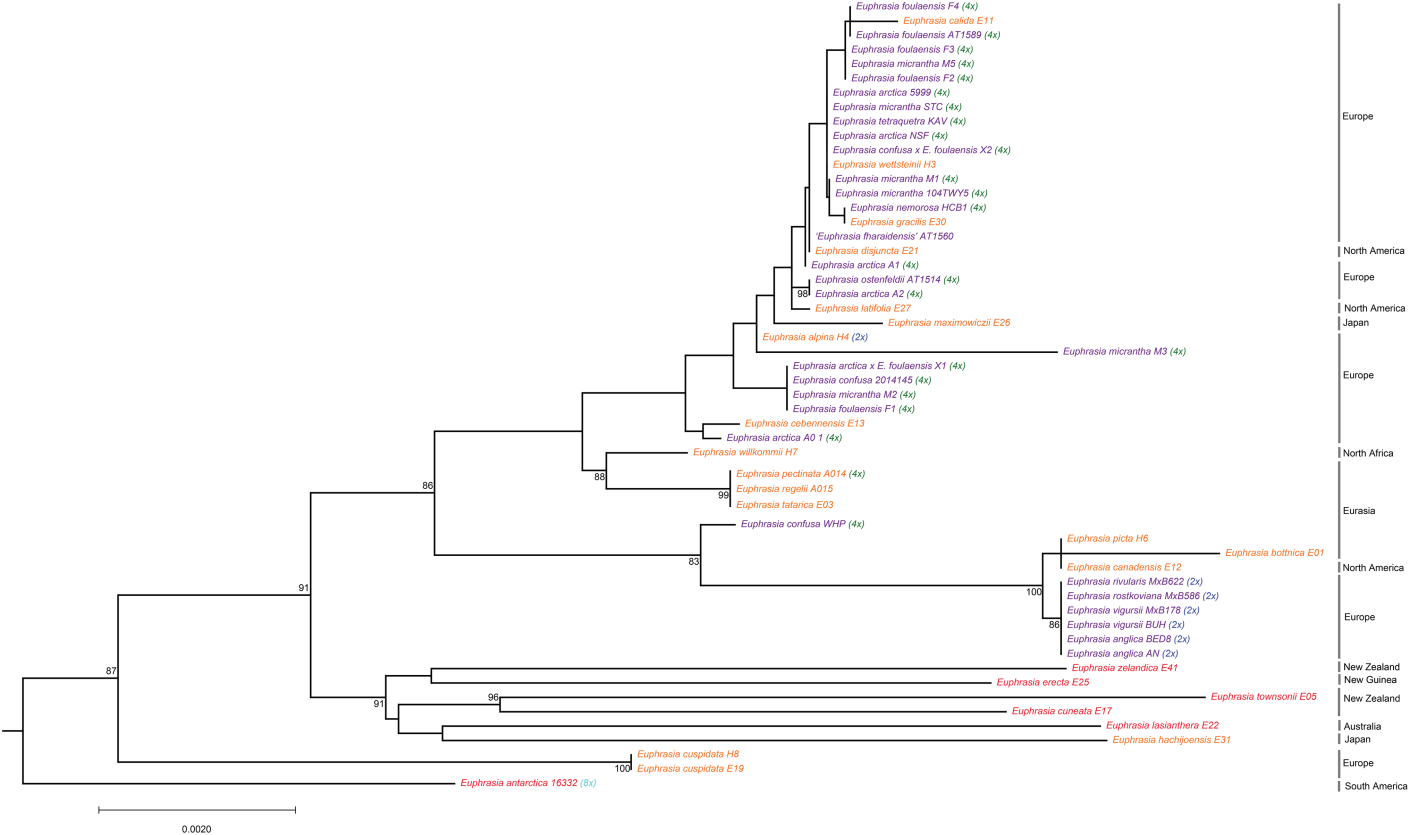
Phylogenetic relationships inferred from nrDNA sequences for global *Euphrasia* species. Phylogeny constructed based on an alignment of 5,845 sites, using a maximum likelihood analysis implemented in IQTree 2. Branches labeled with bootstrap support values over 85%. Phylogeny rooted on *Bartsia*, which is removed here for better visualization. Tip labels are colored and labeled by ploidy, as Figure 1.

Phylogenetic analyses of the nrDNA array confirmed the presence of a clear and moderately well-supported biogeographic break (BS = 86%) largely corresponding to Northern vs. Southern Hemisphere taxa (Figure 2). The southern clade is characterized by long branches typical of older and more divergent lineages, but also reflect artifacts related to poorer sampling. In the Northern Hemisphere clustering is largely by ploidy, with clear separation of most diploid taxa on a well-supported (BS = 100) long branch from most known tetraploids. Within tetraploids, there is a clade comprised of Siberian *E. tatarica* and Chinese *E. regelii* and *E. pectinata*, and a spanish sample of *E. willkommii*. The better-sampled clade of predominantly North Western European tetraploids lacks strong geographic or taxonomic structure, with species and geographic locations being largely intermixed. For example, nrDNA sequences of *E. micrantha* are scattered across the clade and with one sequence on a long branch. However, there is evidence of some geographic structure and clustering by taxonomy, for example with four samples representing three species and one hybrid found on Fair Isle possessing identical nrDNA sequences.

### Nuclear Genome Resequencing Phylogenetic Relationships

**Figure 3:**
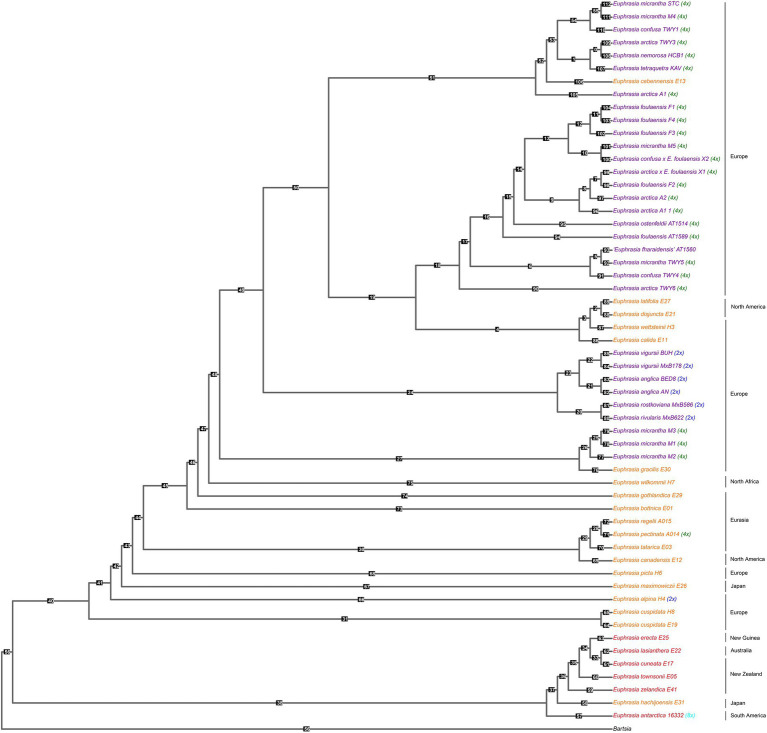
Species tree built from conserved nuclear scaffolds present across *Euphrasia* species. Species tree performed using Astral, based on gene trees for each of 3,454 scaffolds (totaling 46 Mb) generated with IQTree 2. Tips colored by geography and labeled with ploidy as Figure 1. Numbered branch labels correspond to separate bar plots quantifying discordance using Discovista, presented in Figure 4.

**Figure 4:**
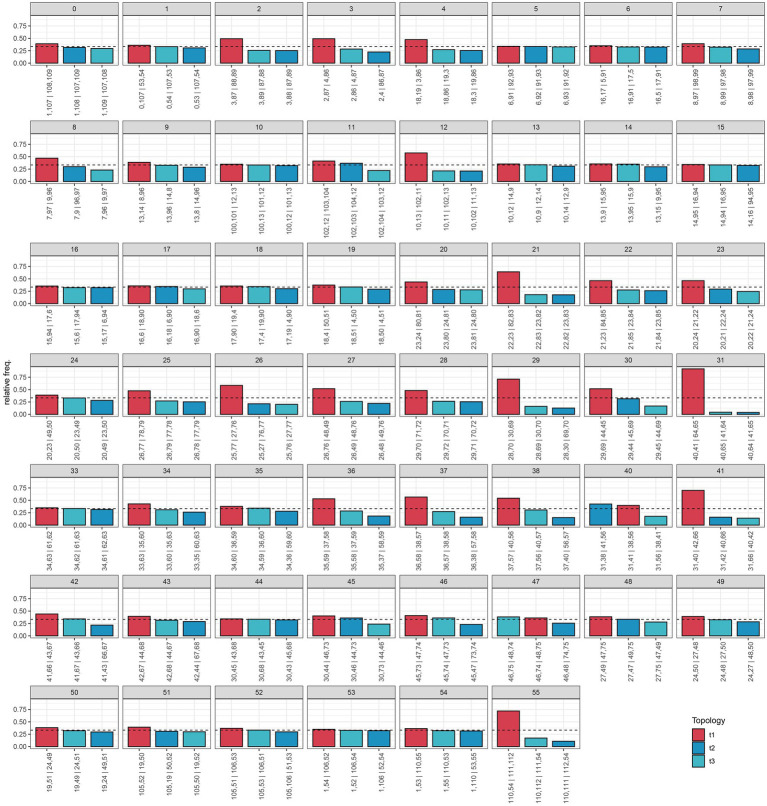
Relative frequencies of alternative tree topologies for *Euphrasia*, computed with DiscoVista. Each bar graph represents potential for the three alternative branching relationships at each focal node labeled in the species tree presented in Figure 3. Main topologies are in red, alternative topologies in blue, and the dotted line indicates a 1/3 threshold (equal representation of three topologies). The x-axis is labeled with neighboring branch labels (see Sayyari et al., 2018).

Our species tree of conserved nuclear scaffolds (Figure 3), and associated quantification of discordance with DiscoVista (Figure 4), revealed that 66% (36/55) of internal nodes had one (and only one) topology across more than 33% of the gene trees. For the remaining (34%) internal branches, there is an equal weighting for a second or third possible topology. Across the species tree, there is notable variation in patterns of discordance. There is generally a lower likelihood of the alternative topology in branches within the clade of Southern Hemisphere taxa and species from Japan (nodes 33–38), which are newly sequenced here, as well as in British diploids (nodes 20–24), or where there is geographically cohesive species sampling of tetraploids (such as three sample of the selfing species *E. foulaensis* sampled on Fair Isle, node 12). In contrast, there is a near equivalent representation of two, or all three, possible relationships in many other branches across the phylogeny, particularly those involving British tetraploids. While the general trend is of greater discordance in branches connecting recent species relationships in European tetraploids, there are also some early diverging nodes with discordance where an alternative topology is frequent (e.g., nodes 40 and 47), showing complexity across the *Euphrasia* phylogeny.

This species tree supports the plastid and nrDNA phylogenies in recovering an early diverging clade predominantly composed of Southern Hemisphere taxa, with the South American taxa placed sister to the Australia-South Asian taxa. The better-sampled Northern Hemisphere clade has the Japanese taxon *E. maximowiczii* included within an otherwise exclusively North American-European clade. Within the Northern Hemisphere clade, there are clearly resolved early diverging relationships between mainland European, Japanese, and North American taxa. British diploid species are resolved as monophyletic, while British tetraploids fall in three large clades, each with a small number of European or North American taxa. Of particular note is one clade where mainland British samples are placed sister to a monophyletic sub-clade of species found on Fair Isle. *Euphrasia micrantha* is found in all three clades including British taxa, including one clade of three *E. micrantha* samples with European *E. gracilis*.

The neighbor-joining tree based on distances estimated by MASH sketches broadly mirrors the topology of the species tree built using trees inferred from the 3,454 conserved sequence scaffolds (Supplementary Figure S1), albeit with some short branches. The only sample showing discordant placement relative to the species tree is *Euphrasia gothlandica* (E29), which suffers from low coverage.

### Comparative Phylogenetics

**Figure 5:**
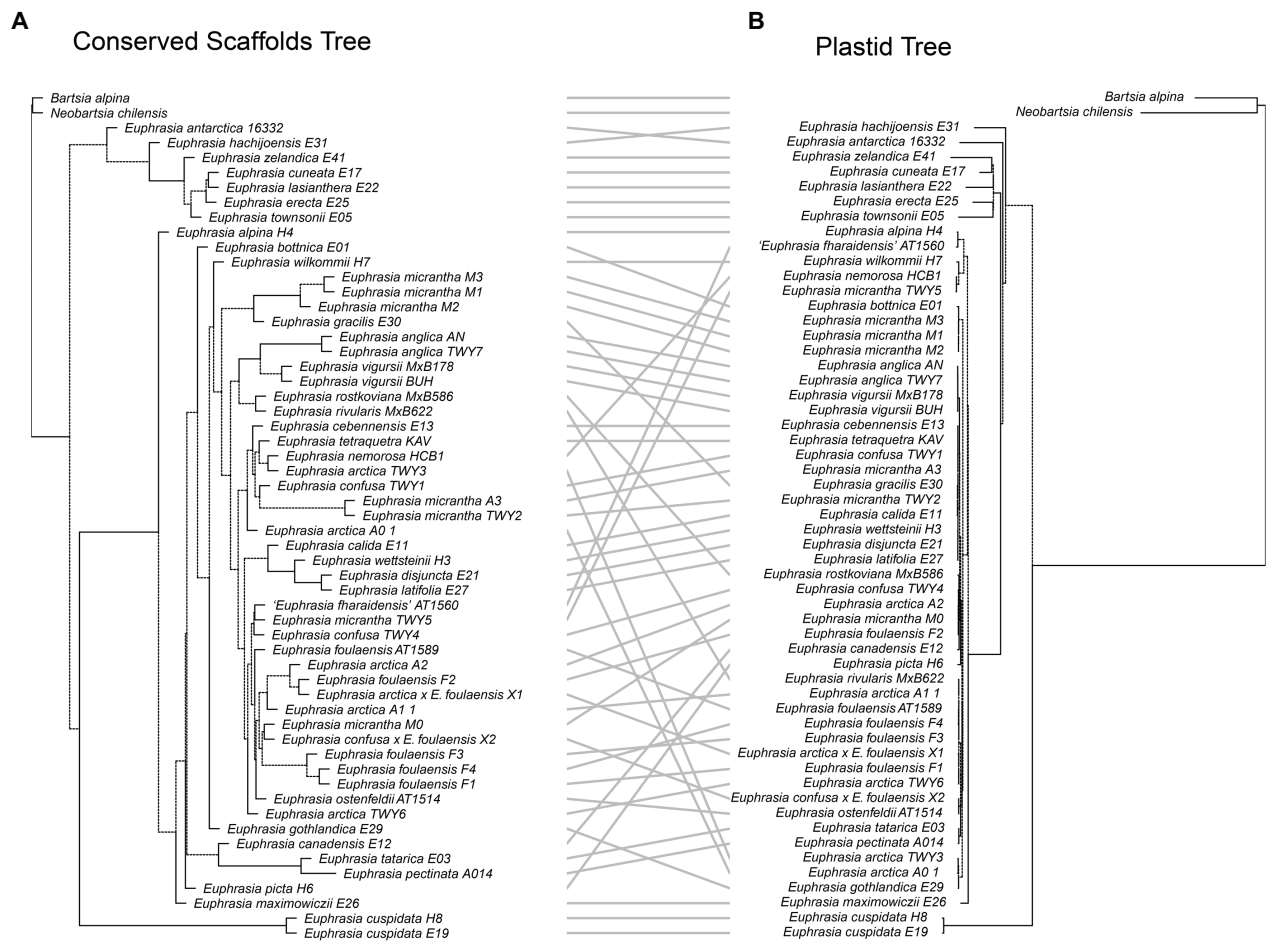
Tanglegram comparing **(A)** the species tree from the conserved nuclear scaffolds and **(B)** the maximum likelihood phylogeny for the plastid genome.

**Figure 6:**
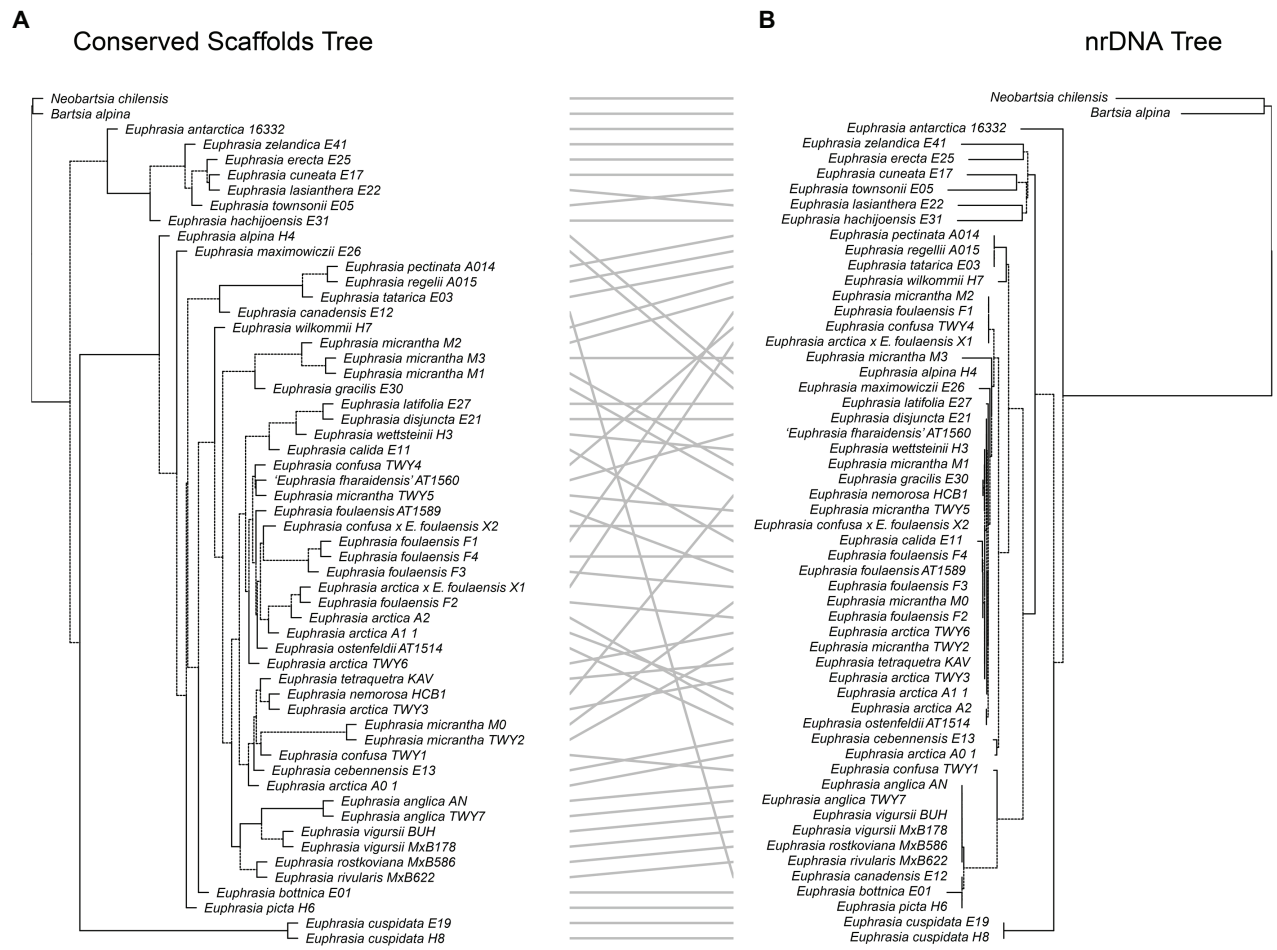
Tanglegram comparing **(A)** the species tree from the conserved nuclear scaffolds and **(B)** the maximum likelihood phylogeny for the nrDNA.

Our tanglegram analyses revealed extensive phylogenetic discordance between genomic regions. Given the large amount of informative sites and high support, we focus on comparisons to the nuclear species tree analysis. Incongruence is seen in comparisons with the plastid genome phylogeny (Figure 5), as the plastid analysis does not recover any major clades apart from the early diverging predominantly Southern Hemisphere group. Most notably, the plastid phylogeny does not recover a group of diploid taxa or resolve any overall geographic structure within the Northern Hemisphere group (Figure 5). When the nuclear species tree is compared to the nrDNA tree (Figure 6), there are some similarities including groups corresponding to ploidy, though its placement within the phylogeny differs, appearing on a long branch sister to all Northern Hemisphere tetraploids in the nrDNA tree, and being placed in a more derived clade in the nuclear species tree. Interestingly however, both the nuclear species tree and the plastid tree have some consistent individual-level relationships, such as three samples from Fair Isle, whereas this group is not recovered in the nrDNA analysis. Otherwise the plastid and nrDNA phylogenies are largely incongruent except some early diverging Southern Hemisphere lineages (Supplementary Figure S2). One interesting case of incongruence is diploid *E. rivularis*, a species of putative cross-ploidy hybrid origin. This is the only diploid species clustering with a group of tetraploids in the plastid tree, but yet it clusters with other diploids in the nrDNA tree, consistent with its proposed origins.

### Genomic Analyses of Polyploidy

We assessed sample ploidy and subgenome relationships based on short-read sequence coverage relative to scaffolds present in the genome of tetraploid *E. arctica*. While the mean mapping depth of ~14X is generally sufficient to infer presence/absence and estimate copy number, there was notable variation, and 5 samples had below 5X mapping which partly obscures patterns (Supplementary Table S2).

**Figure 7:**
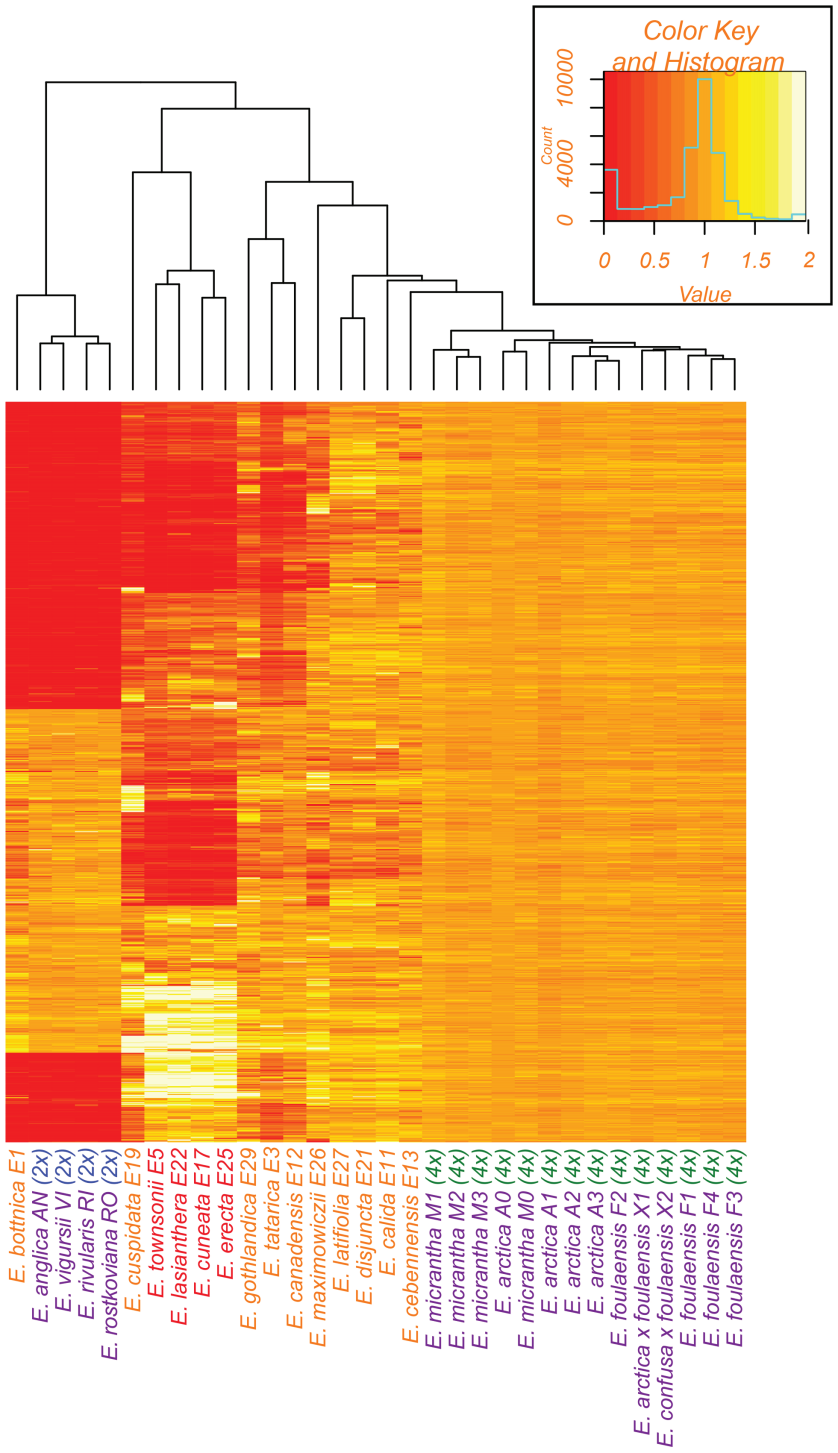
Heatmap of relative mapping depth of *Euphrasia* whole-genome resequencing data relative to the *E. arctica* reference genome. Blocks of color represent mapping coverage (see key, top right) for each scaffold along the y-axis. Species names are colored by geographic area and ploidy as in Figure 1. The tree is based on hierarchical clustering of pairwise Manhattan distances between the samples’ mapping depth profiles.

*Euphrasia bottnica* (E1, Finland) demonstrated a coverage pattern similar to previously analysed British diploids, with a large set of scaffolds having no (or nearly no) reads mapping, with these absent scaffolds corresponding to the divergent tetraploid subgenome (Figure 7). Similarly, *E. calida* (E11, Iceland), *E. cebennensis* (E13, France), *E. disjuncta* (E21, Canada), *E. latifolia* (E27, Canada), and *E. gracilis* (E30, Sweden) show broadly similar coverage patterns to British tetraploids, albeit with higher variance. The other samples had distinctly different mapping depth patterns. However, four samples from the Southern Hemisphere: *E. townsonii* (E5, New Zealand), *E. cuneata* (E17, New Zealand), *E. lasianthera* (E22, Australia), and *E. erecta* (E25, New Guinea), show starkly different coverage patterns for a subset of the conserved scaffolds including both *Euphrasia*-wide conserved scaffolds (3454) and tetraploid-only conserved scaffolds (~7,000). For this subset of scaffolds, these samples show double the average genome coverage, suggesting that these samples are either octoploid or have partial genome duplication post-polyploidization. The inference of polyploid history for samples E3, E12, and E29 is more ambiguous, though these represent tetraploids divergent from the the reference.

## Discussion

Taxonomically complex groups are often neglected in genomic studies in preference of more tractable groups with simpler speciation histories. However, the increasing accessibility of genomic sequencing now make it possible to perform comparative genomic analyses in even the most complex plant groups. Here, we perform a phylogenomic analyses of the renowned taxonomically group *Euphrasia*, with a focus on inferring species cohesion and colonization history of British species, and providing first insights into wider genomic variation present across the genus. Combined, these two approaches allow us to consider the role of polyploidy, geography and species barriers in shaping genome-wide variation. Overall we find extensive phylogenomic discordance at both shallow and deep temporal scales, particularly in comparisons involving the plastid genome. Within the postglacial radiation of *Euphrasia* in northern Europe, we detect discrete waves of colonization to Britain from distinct source populations, with complex patterns of individual relatedness that are generally more closely connected to geographic location than species identity. Across our wider *Euphrasia* analyses, we also see strong geographic structure, as well as clustering by ploidy, indicative of reproductive isolation between diploid and tetraploid taxa. Moreover, comparative analyses of sequencing coverage suggest genomic diversity in *Euphrasia* is a consequence of independent evolutionary radiations of tetraploid species. Here, we consider the implications of these results for understanding speciation processes in this enigmatic group, and more widely for understanding genomic variation across diverse *Euphrasia* species.

### Extensive Phylogenomic Discordance Across the *Euphrasia* Phylogeny

Phylogenetic discordance has been observed in numerous plant studies and is increasingly considered the norm (Rose et al., 2021). Here, we confirm that signals of phylogenetic discordance observed with Sanger sequencing of few loci at the regional (Wang et al., 2018), and the global scale (Gussarova et al., 2008), are indeed detected with genomic data. The lack of clear geographic, taxonomic or ploidy-related structuring in the plastid genome phylogeny, coupled with incongruence to the well-supported nuclear genomic phylogeny, shows the plastid does not track other loci and variation is shaped by other evolutionary forces. In particular, frequent hybridization as well as self-fertilization may lead to the geographically restricted fixation of plastid haplotypes giving a local geographic signal conflicting with species boundaries.

While there is less discordance between nrDNA and the nuclear genome there is still conflict, which may be due to the stochasticity underlying a single gene tree, or a specific outcome of nrDNA being maintained in multiple copies and subsequently experiencing concerted evolution (Xu et al., 2017). Perhaps most importantly, nucleotide ambiguity in the tetraploids suggests the maintenance of multiple nrDNA variants following polyploidy, a finding not observed with direct Sanger sequencing in *Euphrasia* (Wang et al., 2018). While intra-individual variation is problematic for reconstructing phylogenies, the broad concordance of major clades between nrDNA and the nuclear species tree suggests this issue does not obscure signal present in the data. Future work will look to clarify evolutionary relationships using phased nrDNA sequences as has been done with polyploid taxa in the Asteraceae (Fehrer et al., 2021), in the hope that this will reveal the currently unknown second progenitor of British tetraploid *Euphrasia* species (Becher et al., 2020; discussed below).

In addition to challenges with the retention of multiple nrDNA copies, there is also ample evidence consistent with incomplete lineage sorting, with individuals with multiple samples combining variation greatly predating speciation, including variants across the tetraploid clade which was previously estimated to be 7.3 million years old (Gussarova et al., 2008). Regardless of the evolutionary processes shaping variation, these results highlight that an “extended barcode” (Coissac et al., 2016) based on plastid and nrDNA from genome skimming will fail to identify species in *Euphrasia*, as well as a range of other complex groups like willows (Wagner et al., 2021). Here, researchers should instead look to sample the nuclear genome, using methods such as whole-genome resequencing or sequence capture with target enrichment probes (Johnson et al., 2019).

### Speciation and Colonization History

Our analyses used extensive sampling of British species, coupled with representatives from the geographic range and phylogenetic diversity of *Euphrasia*. This allows us to investigate the colonization and speciation history of recent postglacial species divergence. Previous work has shown that diploid and tetraploid *Euphrasia* diverged long before recent pleistocene glaciation events and colonized Britain independently (Wang et al., 2018). Our higher resolution nuclear genomic data further show British tetraploids are present in at least three clades mixed with mainland European samples, and therefore likely represent at least three waves of colonization. These results mirror many other plant phylogeographic studies, where different genetic lineages present in Britain have colonized recurrently from continental Europe and co-exist (Wood et al., 2018).

In addition to broad-scale phylogeographic patterns, our work also reveals fine-scale insights, such as showing that the geographically widespread small-flowered selfing taxon *Euphrasia micrantha* is polyphyletic in analyses of the nuclear genome, plastid and nrDNA array. This finding is surprising, as it is one of the most morphologically distinct *Euphrasia* species, characterized by purple leaves, stems, and flowers, and has a distinctive ecology being predominantly found in heather moorland (Stone, 2012). Our more limited sampling of a number of other taxa reveals a similar lack of species cohesion. These results are in line with previous genetic and genomic studies showing genetic variation in *Euphrasia* often clusters by geography rather than by species, at least within a ploidy level (French et al., 2008; Becher et al., 2020). While phenotypic plasticity and taxonomic confusion are profound challenges for studies of *Euphrasia* (Brown et al., 2020), these are of limited concern at least for *E. micrantha*, which maintains its morphological distinctiveness under a range of conditions and is unlikely to be confused by *Euphrasia* experts. This leaves a number of non-mutually exclusive explanations underlying the origin and maintenance of the species.

Firstly, our analyses here either looked at largely non-recombinant single genomic regions (plastid or nrDNA), or aggregated regions with partially independent evolutionary histories (conserved nuclear scaffolds). In particular the use of scaffolds conserved across individuals means we have only investigated genomic relatedness in one subgenome of the tetraploid. As such these results may have overlooked or masked more subtle genomic signatures at individual nuclear loci. It may be that *E. micrantha* and other *Euphrasia* species are monophyletic at specific nuclear regions underlying species differences that were not analyzed separately here, but experience homogenizing gene flow, such as *via* hybridization across the rest of the genome (Twyford and Friedman, 2015). This remains a distinct possibility given weak reproductive barriers between *Euphrasia* species. Such an explanation would be consistent with either a single origin of the species followed by hybridization, or multiple origins at different sites perhaps from a shared pool of standing genetic variation (i.e., combinatorial speciation *sensu* Marques et al., 2019). However, the maintenance of such high genetic diversity and divergent haplotypes within species, and within a single sampling location, particularly in a selfing taxon, may also point toward the presence of cryptic species. We are currently pursuing these hypotheses using further genomic sequencing of population samples, where we aim to quantify the extent of hybridization between individuals from different geographic areas, and with contrasting ploidy and mating systems.

### Genome Evolution and Polyploidy

The previous study of Becher et al. (2020) identified an allotetraploid origin of British *Euphrasia*, with one subgenome closely related to British diploids. Our analysis of sequencing coverage revealed that *Euphrasia bottnica* sampled from Finland possesses a similar genome to extant British diploids, and this species clusters with diploids in the nrDNA phylogeny, suggesting a shared genomic affinity across this region of postglacial recolonization. Similarly, a number of European tetraploid taxa, such as *E. calida* and *E. cebennensis*, as well as Canadian *E. disjuncta* and *E. latifolia*, have similar sequence coverage patterns to British tetraploids, consistent with a shared allopolyploid origin for these Northern Hemisphere taxa. Perhaps more notable are cases such as *E. cuneata* and *E. townsonii* (New Zealand), *E. lasianthera* (Australia), *E. erecta* (New Guinea), and *E. cuspidata* (Austria), which are characterized by an extremely different coverage patterns, with some scaffolds in the *E. arctica* reference having no coverage and others having two-fold coverage. Firm conclusions of the ploidy and subgenome constituents of these taxa are hard to make given they are likely to show substantial divergence from the British *E. arctica* reference genome, and are also characterized by low mapping coverage. We have attempted to further characterize sequence reads from these individuals using k-mer based approaches including KAT and Tetmer (Mapleson et al., 2017; Becher et al., 2020), but failed to retrieve a clear signal (Unpublished Results), likely due to low sequencing coverage and potential DNA degradation from these herbarium specimens. Regardless, the finding that these divergent species possess a different genome structure to other *Euphrasia* warrants further study, and suggests recurrent polyploidy in the genus, in line with known tetraploids and hexaploids being scattered across the *Euphrasia* phylogeny.

### Prospect of Phylogenomic Analyses in Taxonomically Complex Groups

Taxonomically complex groups frequently pose the joint challenges of taxonomic issues, where species definitions may be uncertain and monographic work is often sorely needed, and systematic/phylogenetic issues, where molecular phylogenies show complex patterns of relatedness. Both issues are relevant to *Euphrasia*. Much has been learnt about species limits since the world monograph of the genus by von Wettstein (1896) and the European revision by Yeo (1978), not least the extent of phenotypic plasticity that taxa exhibit in response to host species and ecological conditions (Karlsson, 1984; Zopfi, 1997; Brown et al., 2020). Our general view is that *Euphrasia* species as currently described, particularly in Britain, may have been too finely divided, and future monographic work may look to “lump” a range of species where trait differences are minimal or prove unreliable. Despite these taxonomic issues, we note that even the most distinct species, such as *E. micrantha*, are not monophyletic (discussed above), showing phylogenetic complexity will persist even following taxonomic realignment of species. This is unsurprising given the nature of these species, showing recent speciation, rampant hybridization, and selfing or mixed-mating systems.

One source of samples that has proved particularly useful in our study has been verified material present in herbarium collections. The search for genomic tools that reliably recover information from herbarium specimens is driven by the incredible amount of historic plant diversity contained within these collections (Särkinen et al., 2012; Buerki and Baker, 2016; Brewer et al., 2019). Accessing herbaria’s genomic data will allow researchers to (figuratively) travel through time and space to study extinct taxa and changes in genetic diversity over time. Many studies have now demonstrated the efficacy of genome skimming or target capture to recover genomic data from historical samples (Zeng et al., 2018). Both these approaches rely on a form of “enrichment,” with genome skimming analyzing “naturally enriched” regions (i.e., those at high copy number) while target capture enriches regions homologous to target baits. Here, we show that non-enriched, direct whole-genome sequencing can be successfully used for degraded herbarium material. As well as being used to assemble the plastid and nrDNA array, we were able to map sufficient reads to the *E. arctica* reference genome to infer sample ploidy. While useful, however, there were notable issues with these analyses, particularly due to low sample mapping depths. This may either be a consequence of species divergence or contamination, with previous work showing over 70% of sequence reads from herbarium material may be contaminants (Bieker et al., 2020). This issue, combined with DNA error profiles of dried plant tissue, prevented us performing further characterization of these genomes, and suggests future work must oversample herbarium DNA to ensure sufficient data post bioinformatic filtering, or use silica dried plant tissue where available (Brewer et al., 2019). Despite these concerns, we found neighbor-joining trees generated from raw sequence reads from herbarium samples, inferred using MASH, mirrored the topology of our more rigorous scaffold-based nuclear phylogenetic analyses, suggesting sample degradation and contamination do not obscure the main signal of genome-wide relatedness.

Phylogenomic analyses of taxonomically complex groups are often made difficult due to reticulation coupled with polyploidy. Here, we circumvented a number of issues by analyzing haploid plastid genomes, though our hope that nrDNA would have been homogenized within an individual appears not to be the case. We similarly focused our nuclear genome analyses on conserved disomically inherited scaffolds, allowing us to compare across diverse ploidies and to represent evolutionary relationships using a species tree analysis. We chose not to further interrogate genomic relationships within putative subgenomes due to the uncertain homology across these diverse species of differing ploidy and with potentially different parental progenitors. Future work in *Euphrasia*, and other taxonomically complex groups, may look to long-read sequencing and pangenome analyses to better represent structural genomic variation across diverse taxa without reference bias, and to provide robust sorting of homoeologs between subgenomes (Bayer et al., 2021). More integrated polyploidy-aware phylogenomic networks, such as alloPPnet, are also likely to prove fruitful, particularly in the future if this or other methods are developed that are less computationally demanding and allow larger multi-sample data sets as well as more diverse ploidy levels (Rothfels, 2021).

### Conclusion

Studies of the extent of discordance in phylogenies have given important insights into a range of topics, including hybridization (Patterson et al., 2012; Martin et al., 2013) and hybrid speciation (Köhler et al., 2021), evolutionary conflict (Hedtke and Hillis, 2010), horizontal gene transfer (Davis et al., 2005) and rates of phenotypic innovation (Parins-Fukuchi et al., 2021). Our study shows taxonomically complex *Euphrasia* represent a genus where phylogenetic discordance is extensive, at both shallow and deep nodes in the phylogeny. This discordance is likely to be driven by the interaction of different processes, including recurrent rounds of polyploidy, rampant hybridization, and recent postglacial species divergence. Future work will look to estimate the contribution of these processes to phylogenetic conflict in chromosome level genome assemblies.

## Data Availability Statement

The newly generated raw sequence reads are available in the SRA, and plastid genomes and nrDNA sequences are in Genbank. The sequence alignments, phylogenetic trees and scripts for Tanglegrams are provided in Dryad (https://doi.org/10.5061/dryad.jh9w0vtd).

## Author Contributions

PG, AT, CP, and RN designed the research. GG and AT provided samples. PG generated sequencing data. PG, HB, SG, and AT analyzed the data. PG and AT wrote the first draft of the manuscript. All authors contributed to the article and approved the submitted version.

## Funding

This work was funded by the NERC International Opportunities Fund Grant NE/N006739/1 “Evolutionary consequences of facultative plant parasitism” awarded to AT, GG, CP, and RN, and grants NE/R010609/1 and NE/L011336/1 awarded to AT. Plant.ID has received funding from the European Union’s Horizon 2020 research and innovation program under the Marie Skłodowska-Curie grant agreement No. 765000. The Royal Botanic Garden Edinburgh is supported by the Scottish Government’s Rural and Environmental Science and Analytical Services Division.

## Conflict of Interest

The authors declare that the research was conducted in the absence of any commercial or financial relationships that could be construed as a potential conflict of interest.

## Publisher’s Note

All claims expressed in this article are solely those of the authors and do not necessarily represent those of their affiliated organizations, or those of the publisher, the editors and the reviewers. Any product that may be evaluated in this article, or claim that may be made by its manufacturer, is not guaranteed or endorsed by the publisher.
