# Supplementary material for: Pervasive Phylogenomic Incongruence Underlies Evolutionary Relationships in Eyebrights (Euphrasia, Orobanchaceae)
Source: Front Plant Sci. 2022 May 27;13:869583. doi: 10.3389/fpls.2022.869583 (PMC9197813; doi:10.3389/fpls.2022.869583)
Supplement: Supplementary file 1 [file Data_Sheet_1.pdf]

Supplementary Material

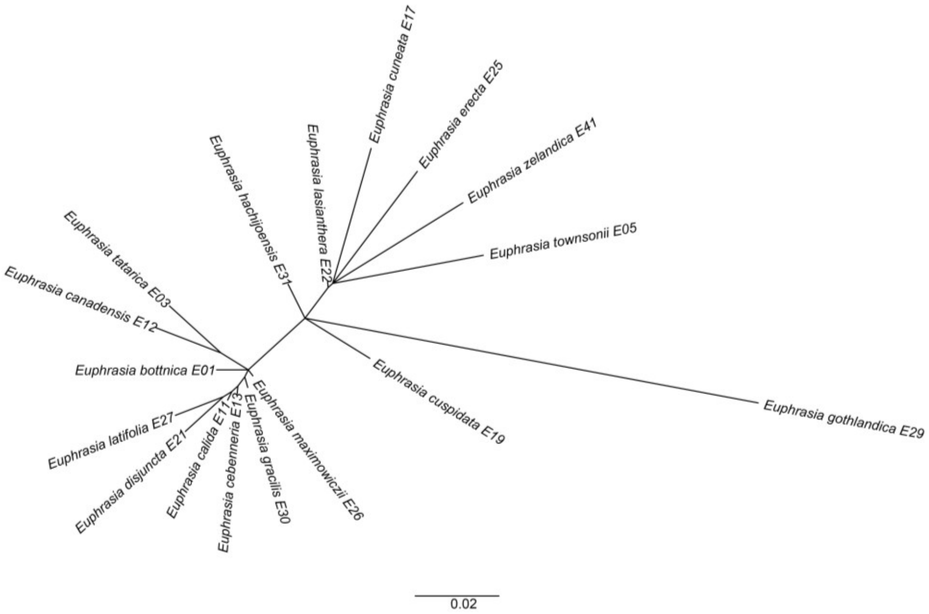

Figure S1. Unrooted neighbour joining tree for 17 *Euphrasia* herbarium specimens based on MASH distances from Illumina sequence reads.

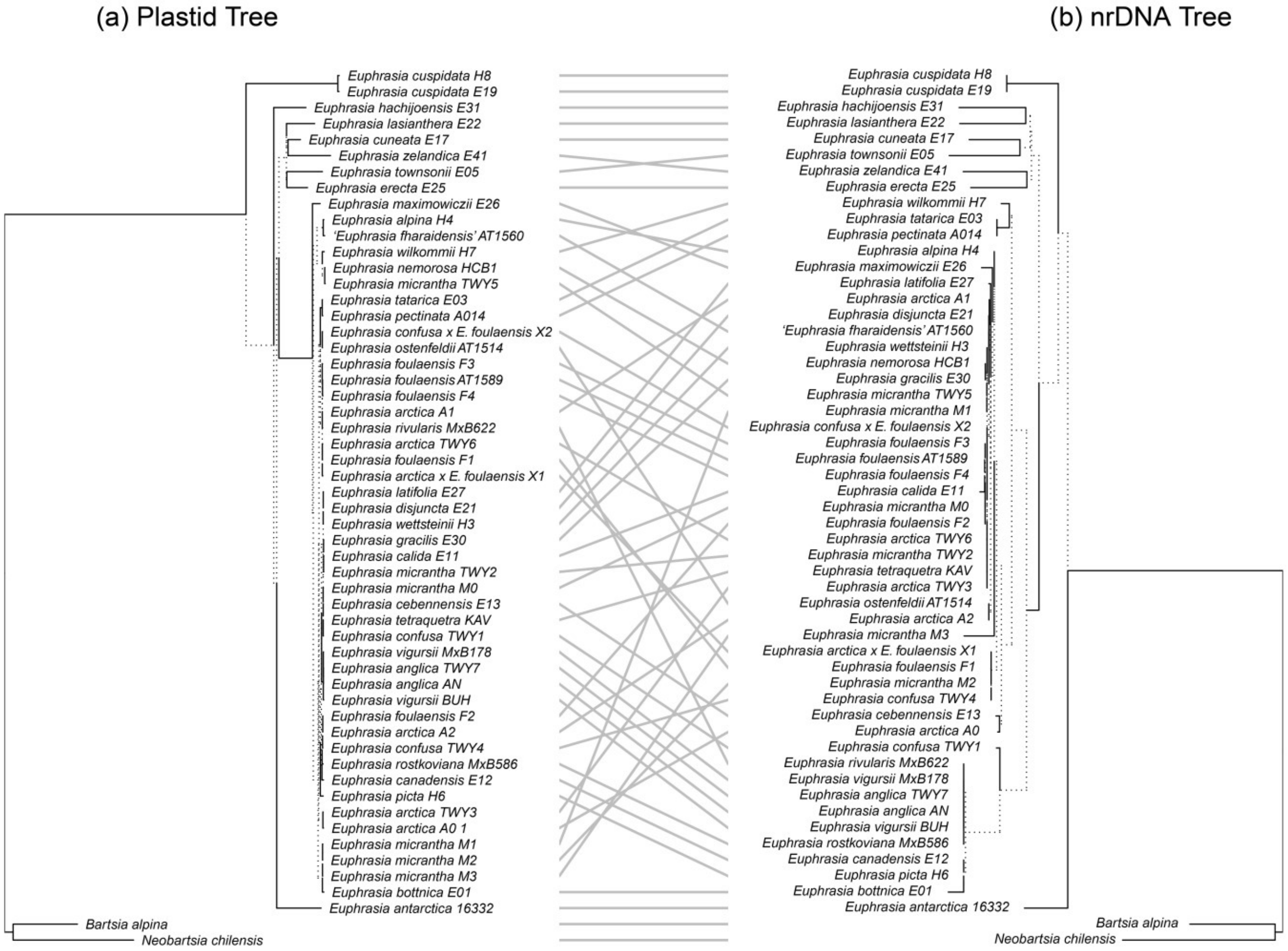

Figure S2. Tanglegram comparing (a) the maximum likelihood phylogeny for the plastid genome (b) the maximum likelihood phylogeny for nrDNA.

Table S1. Sample accession information for *Euphrasia* and outgroup samples.

Sample ID	Literature ploidy	Alternative ID numbers	Herbarium collection year	Geographic location	Geographic area	Reference
Bartsia alpina 11875			n/a	Faroe Islands	Europe	Becher et al. 2021
Euphrasia alpina H4	diploid	E012	n/a	Spain	Europe	Twyford Unpublished
Euphrasia anglica AN	diploid	E030	n/a	Britain	UK	Becher et al. 2020
Euphrasia anglica TWY7	diploid	E001 / BED8	n/a	Britain	UK	Becher et al. 2021
Euphrasia antarctica 16332	octoploid	E008	n/a	South America	South America	Twyford Unpublished
Euphrasia arctica A0	tetraploid	E028	n/a	Britain	UK	Becher et al. 2020
Euphrasia arctica A1	tetraploid	E027	n/a	Britain	UK	Becher et al. 2020
Euphrasia arctica A2	tetraploid	E026	n/a	Britain	UK	Becher et al. 2020
Euphrasia arctica A3	tetraploid		n/a	Britain	UK	Becher et al. 2020
Euphrasia arctica TWY3	tetraploid	E005	n/a	Britain	UK	Becher et al. 2021
Euphrasia arctica TWY6	tetraploid	E002	n/a	Britain	UK	Becher et al. 2021
Euphrasia arctica x foulaensis X1	tetraploid	E034	n/a	Britain	UK	Becher et al. 2020
Euphrasia bottnica E01			1979	Finland	Europe	This study
Euphrasia calida E11			1934	Iceland	Europe	This study

Euphrasia canadensis E12			1992	Canada	North America	This study
Euphrasia cebennensis E13			1861	France	Europe	This study
Euphrasia confusa TWY1	tetraploid	E007	n/a	Britain	UK	Twyford Unpublished
Euphrasia confusa TWY4	tetraploid	E004	n/a	Britain	UK	Twyford Unpublished
Euphrasia confusa x foulaensis X2	tetraploid	E039	n/a	Britain	UK	Becher et al. 2020
Euphrasia cuneata E17			1972	New Zealand	Australasia	This study
Euphrasia cuspidata E19			1973	Austria	Europe	This study
Euphrasia cuspidata H8		E009	n/a	Europe	Europe	Becher et al. 2021
Euphrasia disjuncta E21			1949	Canada	North America	This study
Euphrasia erecta E25			1965	New Guinea	Australasia	This study
'Euphrasia fharaidensis' AT1560		E017	n/a	Britain	UK	Becher et al. 2021
Euphrasia foulaensis AT1589	tetraploid	E018	n/a	Britain	UK	Becher et al. 2021
Euphrasia foulaensis F1	tetraploid	E025	n/a	Britain	UK	Becher et al. 2020
Euphrasia foulaensis F2	tetraploid	E024	n/a	Britain	UK	Becher et al. 2020
Euphrasia foulaensis F3	tetraploid	E038	n/a	Britain	UK	Becher et al. 2020

Euphrasia foulaensis F4	tetraploid	E037	n/a	Britain	UK	Becher et al. 2020
Euphrasia gothlandica E29			Unknown	Sweden	Europe	This study
Euphrasia gracilis E30			1894	Sweden	Europe	This study
Euphrasia hachijoensis E31			1954	Japan	Asia	This study
Euphrasia lasianthera E22			1971	Australia	Australasia	This study
Euphrasia latifolia E27			1910	Canada	North America	This study
Euphrasia maximowiczii E26			1997	Japan	Asia	This study
Euphrasia micrantha M0	tetraploid		n/a	Britain	UK	Becher et al. 2020
Euphrasia micrantha M1	tetraploid	E023	n/a	Britain	UK	Becher et al. 2020
Euphrasia micrantha M2	tetraploid	E022	n/a	Britain	UK	Becher et al. 2020
Euphrasia micrantha M3	tetraploid	E035	n/a	Britain	UK	Becher et al. 2020
Euphrasia micrantha TWY2	tetraploid	E006 / STC	n/a	Britain	UK	Becher et al. 2021
Euphrasia micrantha TWY5	tetraploid	E003	n/a	Britain	UK	Becher et al. 2021
Euphrasia nemorosa HCB1	tetraploid	E016	n/a	Britain	UK	Becher et al. 2021

Euphrasia ostenfeldii AT1514	tetraploid	E019	n/a	Britain	UK	Becher et al. 2021
Euphrasia pectinata A014	tetraploid	E014	n/a	China	Asia	Twyford Unpublished
Euphrasia picta H6	diploid	E011	n/a	Austria	Europe	Twyford Unpublished
Euphrasia regelii A015		E015	n/a	China	Asia	Twyford Unpublished
Euphrasia rivularis MxB622	diploid	E032	n/a	Britain	UK	Becher et al. 2020
Euphrasia tatarica E03			1914	Siberia	Europe	This study
Euphrasia tetraquetra KAV	tetraploid	E020	n/a	Britain	UK	Twyford Unpublished
Euphrasia townsonii E05			1912	New Zealand	Australasia	This study
Euphrasia vigursii BUH	diploid	E021	n/a	Britain	UK	Becher et al. 2021
Euphrasia vigursii MxB178	diploid	E031	n/a	Britain	UK	Becher et al. 2020
Euphrasia wettsteinii H3	tetraploid	E013	n/a	Northern Europe	Europe	Twyford Unpublished
Euphrasia wilkommii H7		E010	n/a	North Africa	North Africa	Twyford Unpublished
Euphrasia zelandica E41			1983	New Zealand	Australasia	This study
Neobartsia chilensis HB			n/a	Chile	South America	Twyford Unpublished

Table S2. Sequencing summary statistics for newly sequenced *Euphrasia* herbarium samples. [a]Total number of reads (2X number of read pairs), [b]Count of reads post-read merging and the removal of low quality reads, [c]Count of reads mapping to the *E. arctica* genome, [d]Count of reads uniquely mapping to the reference genome.

Species	Collection code	Total reads [a]	Reads retained post-filtering [b]	% retained	Reads mapped [c]	% mapped	Reads uniquely mapped[d]	% uniquely mapped	Coverage[e]
Euphrasia bottnica	E01	276,566,606	210,975,010	77	139,116,411	66	103,961,611	49	24.1
Euphrasia tatarica	E03	268,518,234	191,350,067	72	87,629,661	46	45,529,803	24	10.7
Euphrasia townsonii	E05	265,716,788	188,055,662	71	18,846,190	10	9,517,918	5	2.3
Euphrasia calida	E11	263,154,186	187,012,005	72	133,612,217	71	94,413,746	50	22.7
Euphrasia canadensis	E12	274,709,392	242,396,830	89	175,363,613	72	130,054,027	54	28.4
Euphrasia cebennensis	E13	258,045,248	184,957,673	72	118,634,066	64	89,646,082	48	21.7
Euphrasia cuneata	E17	261,462,248	199,731,933	77	22,307,933	11	13,702,534	7	3.1
Euphrasia cuspidata	E19	311,169,314	263,729,335	85	132,813,087	50	93,671,657	36	20.4
Euphrasia disjuncta	E21	312,518,834	232,838,617	75	163,780,407	70	112,384,839	48	26.6
Euphrasia lasianthera	E22	299,784,118	238,168,427	80	46,778,123	20	31,078,682	13	6.9
Euphrasia erecta	E25	255,642,728	176,142,665	69	16,702,068	9	10,852,617	6	2.5
Euphrasia maximowiczii	E26	278,776,030	217,570,011	79	148,309,876	68	101,375,871	47	23.1
Euphrasia latifolia	E27	276,544,058	206,369,545	75	147,125,676	71	109,019,070	53	25.6
Euphrasia gothlandica	E29	280,015,252	243,503,307	87	4,977,933	2	2,811,716	1	0.6
Euphrasia gracilis	E30	295,825,610	218,205,199	74	93,997,474	43	64,710,286	30	15.5
Euphrasia hachijoensis	E31	241,730,838	157,678,609	66	14,635,661	9	7,195,972	5	1.7

Euphrasia zelandica	E41	285,847,208	231,503,601	81	36,569,619	16	22,746,231	10	5.1
